# Supplementary material for: FLASH-MM: fast and scalable single-cell differential expression analysis using linear mixed-effects models
Source: Nat Commun. 2026 Feb 5;17:2384. doi: 10.1038/s41467-026-69063-2 (PMC12982622; doi:10.1038/s41467-026-69063-2)
Supplement: Supplementary file 2 — Reporting Summary [file 41467_2026_69063_MOESM2_ESM.pdf]

nature portfolio

Corresponding author(s): Gary Bader

Last updated by author(s): Dec 10, 2025

Reporting Summary

Nature Portfolio wishes to improve the reproducibility of the work that we publish. This form provides structure for consistency and transparency in reporting. For further information on Nature Portfolio policies, see our Editorial Policies and the Editorial Policy Checklist.

Statistics

For all statistical analyses, confirm that the following items are present in the figure legend, table legend, main text, or Methods section.

n/a	Confirmed	
☐	☒	The exact sample size (*n*) for each experimental group/condition, given as a discrete number and unit of measurement
☐	☒	A statement on whether measurements were taken from distinct samples or whether the same sample was measured repeatedly
☐	☒	The statistical test(s) used AND whether they are one- or two-sided<br>*Only common tests should be described solely by name; describe more complex techniques in the Methods section.*
☐	☒	A description of all covariates tested
☐	☒	A description of any assumptions or corrections, such as tests of normality and adjustment for multiple comparisons
☐	☒	A full description of the statistical parameters including central tendency (e.g. means) or other basic estimates (e.g. regression coefficient) AND variation (e.g. standard deviation) or associated estimates of uncertainty (e.g. confidence intervals)
☐	☒	For null hypothesis testing, the test statistic (e.g. *F*, *t*, *r*) with confidence intervals, effect sizes, degrees of freedom and *P* value noted<br>*Give P values as exact values whenever suitable.*
☒	☐	For Bayesian analysis, information on the choice of priors and Markov chain Monte Carlo settings
☐	☒	For hierarchical and complex designs, identification of the appropriate level for tests and full reporting of outcomes
☐	☒	Estimates of effect sizes (e.g. Cohen's *d*, Pearson's *r*), indicating how they were calculated

Our web collection on statistics for biologists contains articles on many of the points above.

Software and code

Policy information about availability of computer code

Data collection	No data was collected
Data analysis	The FLASH-MM software is openly available as an R package, including example case scenarios, at the following repositories:<br>https://cran.r-project.org/web/packages/FLASHMM/index.html<br>https://github.com/BaderLab/FLASHMM<br>The package is distributed under the MIT License. Analysis scripts used for case studies are available at: https://github.com/BaderLab/FLASH-MM-analysis/<br><br>Software and R packages used for data analysis:<br>R (v4.4.1); RStudio (v2024.12.0+467);<br>FLASHMM (v1.2.1);<br>lme4 (v1.1-37); lmerTest (v3.1-3); MASS (v7.3-65); Matrix (v1.7-3);<br>Seurat (v5.1.0); SingleCellExperiment (v1.27.2);<br>ggplot2 (v3.5.1); ggrepel (v0.9.6); RColorBrewer (v1.1-3); viridis (v0.6.5); ggpubr (v0.6.0);<br>dplyr (v1.1.4); plyr (v1.8.9); scales (v1.3.0);<br>gprofiler2 (v0.2.3).

For manuscripts utilizing custom algorithms or software that are central to the research but not yet described in published literature, software must be made available to editors and reviewers. We strongly encourage code deposition in a community repository (e.g. GitHub). See the Nature Portfolio guidelines for submitting code & software for further information.

Data

Policy information about availability of data

All manuscripts must include a data availability statement. This statement should provide the following information, where applicable:

- Accession codes, unique identifiers, or web links for publicly available datasets
- A description of any restrictions on data availability
- For clinical datasets or third party data, please ensure that the statement adheres to our policy

The healthy human kidney atlas18 data files were downloaded from the UCSC Cell Browser at https://cells.ucsc.edu/?ds=living-donor-kidney. The Tuberculosis (TB) memory T cell dataset19 can be accessed from the GEO with accession code GSE158769 [https://www.ncbi.nlm.nih.gov/geo/query/acc.cgi?acc=GSE158769]. The stimulated PBMC data16 was downloaded from muscData package (Kang18_8vs8) at https://github.com/HelenaLC/muscData. Source data is provided as a Source Data file.

Research involving human participants, their data, or biological material

Policy information about studies with human participants or human data. See also policy information about sex, gender (identity/presentation), and sexual orientation and race, ethnicity and racism.

Reporting on sex and gender	No original data has been collected in this study. Sex-based variation has been evaluated in a single cell kidney map, using the metadata collected by the original study.
Reporting on race, ethnicity, or other socially relevant groupings	Not applicable.
Population characteristics	Metadata associated with the TB single cell atlas includes donor identity, sex, batch, cluster annotations, and TB status. The dataset includes cells from 259 unique donors, spanning 46 batches and 29 cell clusters. The healthy human kidney transcriptomic map was generated from 27,677 cells obtained from 19 living donors (10 female and 9 male).
Recruitment	Not applicable.
Ethics oversight	Not applicable.

Note that full information on the approval of the study protocol must also be provided in the manuscript.

Field-specific reporting

Please select the one below that is the best fit for your research. If you are not sure, read the appropriate sections before making your selection.

[x] Life sciences [ ] Behavioural & social sciences [ ] Ecological, evolutionary & environmental sciences

For a reference copy of the document with all sections, see nature.com/documents/nr-reporting-summary-flat.pdf

Life sciences study design

All studies must disclose on these points even when the disclosure is negative.

Sample size	The healthy human kidney transcriptomic map was generated from 27,677 cells obtained from 19 living donors (10 female and 9 male).<br>The Tuberculosis (TB) memory T cell dataset was obtained from Nathan et al., 2021, comprising 500,089 cells. The raw count matrix was used for preprocessing. Metadata associated with the cells includes donor identity, sex, batch, cluster annotations, and TB status. The dataset includes cells from 259 unique donors, spanning 46 batches and 29 cell clusters. In the DE analysis, we modeled the donors as a random effect and ignored the batch effect because the majority of donors were sequenced in a single batch.<br>FLASH-MM is a statistical method. We determined the number of datasets based on the need to demonstrate both biological applicability and scalability. One moderately sized atlas (healthy kidney, 27,677 cells from 19 donors) and one large cohort dataset (TB memory T cells, ~500K cells from 259 donors) were selected to showcase performance across realistic study designs. Model accuracy, false-positive control, and scalability were extensively validated through simulation studies (Fig. 2), ensuring that these datasets are sufficient to establish method performance in practical applications.
Data exclusions	The Tuberculosis (TB) memory T cell dataset: Pre-processing steps consisted of removing cells and genes to remove extreme values. Library sizes were assessed using a boxplot, and cells with library sizes outside the lower whisker and above an upper threshold of 2^15 were removed, resulting in the retention of 499,973 cells. Genes were filtered in two steps: first, genes expressed in fewer than 2^9 cells (512 cells) were excluded; second, genes with a counts-per-cell ratio less than 0.005 (i.e., total gene count divided by the number of cells below 0.5%) were removed. These filtering steps reduced the dataset to 11,596 genes and 499,973 cells, which were used for downstream analyses.<br>The healthy human kidney transcriptomic map: Cells were filtered based on three criteria: the number of detected features (nFeature), library size, and the number of cells within each cell type (cluster). The number of features per cell, defined as the total number of non-zero genes, was required to meet a minimum threshold of 100. Library size, calculated as the total counts per cell, was restricted to a range of 2^9 to 2^16. Cell types with fewer than 20 cells were excluded, and “Podocyte” cells were removed due to their low sample size (16 cells post filtering). Additionally, genes were filtered based on their expression levels, where the counts per cell ratio had to exceed 0.005 (i.e., the total gene count divided by the number of cells had to be greater than 0.5%). Genes were further filtered to retain those expressed in at least 16

	cells, with a minimum of 10 cells in each group, total counts between 2^6 and 2^20, and a counts per cell ratio above the threshold. After these filtering steps, 27,550 cells and a refined set of genes were retained for downstream analyses.
Replication	For the biological applications, each donor is treated as an independent replicate. The healthy kidney dataset includes 19 donors, and the TB memory T-cell dataset includes 259 donors. These are included as random effects in the FLASHMM model.<br>Also, simulation experiments were repeated across multiple independently generated datasets. Supplementary QQ-plots demonstrate that FLASH-MM controls false positives under the null hypothesis across six different sample sizes. Supplementary ROC curves show consistent true-positive and false-positive performance across these setups.
Randomization	No new samples were allocated by the investigators. Group labels (sex in the kidney dataset; TB status in the T-cell dataset) were predefined in the original studies. Because allocation was not randomized, covariates were controlled statistically using linear mixed-effects models. Cell type was included as a fixed effect in both analyses, and its interaction with the covariate of interest (sex or TB status) enabled testing differential effects within each cell type. Individual variability was controlled by modeling donor/sample identity as a random effect. The model formulas were:<br>Kidney:<br>~ log(library.size) + Cell_Types_Broad + Cell_Types_Broad:sex + (1|sampleID)<br>TB:<br>~ log(library.size) + cluster_name + cluster_name:TB_status + (1|donor)
Blinding	Blinding was not relevant to this study. All analyses were performed on previously generated, publicly available single-cell RNA-seq datasets with fixed metadata annotations (e.g., donor, sex, disease status). No new data were collected, and all analyses were fully computational.

Reporting for specific materials, systems and methods

We require information from authors about some types of materials, experimental systems and methods used in many studies. Here, indicate whether each material, system or method listed is relevant to your study. If you are not sure if a list item applies to your research, read the appropriate section before selecting a response.

Materials & experimental systems

n/a	Involved in the study
☒	☐ Antibodies
☒	☐ Eukaryotic cell lines
☒	☐ Palaeontology and archaeology
☒	☐ Animals and other organisms
☒	☐ Clinical data
☒	☐ Dual use research of concern
☒	☐ Plants

Methods

n/a	Involved in the study
☒	☐ ChIP-seq
☒	☐ Flow cytometry
☒	☐ MRI-based neuroimaging

Plants

Seed stocks	*Report on the source of all seed stocks or other plant material used. If applicable, state the seed stock centre and catalogue number. If plant specimens were collected from the field, describe the collection location, date and sampling procedures.*
Novel plant genotypes	*Describe the methods by which all novel plant genotypes were produced. This includes those generated by transgenic approaches, gene editing, chemical/radiation-based mutagenesis and hybridization. For transgenic lines, describe the transformation method, the number of independent lines analyzed and the generation upon which experiments were performed. For gene-edited lines, describe the editor used, the endogenous sequence targeted for editing, the targeting guide RNA sequence (if applicable) and how the editor was applied.*
Authentication	*Describe any authentication procedures for each seed stock used or novel genotype generated. Describe any experiments used to assess the effect of a mutation and, where applicable, how potential secondary effects (e.g. second site T-DNA insertions, mosiacism, off-target gene editing) were examined.*
